# Trends in Use of Granulocyte Colony-Stimulating Factor Following Introduction of Biosimilars Among Adults With Cancer and Commercial or Medicare Insurance From 2014 to 2019

**DOI:** 10.1001/jamanetworkopen.2021.33474

**Published:** 2021-11-23

**Authors:** Ching-Yu Wang, Coy D. Heldermon, Scott M. Vouri, Haesuk Park, Sarah E. Wheeler, Brian Hemendra Ramnaraign, Nam Hoang Dang, Joshua D. Brown

**Affiliations:** 1 Department of Pharmaceutical Outcomes and Policy, College of Pharmacy, University of Florida, Gainesville; 2 Center for Drug Evaluation and Safety, University of Florida, Gainesville; 3 College of Medicine, University of Florida, Gainesville; 4 Department of Pharmaceutical Services, University of Florida Health Shands Cancer Hospital, Gainesville; 5 University of Florida Health Cancer Center, Gainesville; 6 Division of Hematology and Oncology, Department of Medicine, University of Florida, Gainesville

## Abstract

**Importance:**

The introduction of biosimilars and novel delivery devices between 2014 and 2019 may have changed the utilization of granulocyte colony-stimulating factors (G-CSF).

**Objective:**

To assess utilization trends of G-CSFs for primary prophylaxis of febrile neutropenia (FN) among patients with cancer receiving myelosuppressive chemotherapy with commercial or Medicare insurance.

**Design, Setting, and Participants:**

This cross-sectional study assessed G-CSF utilization trends overall and stratified by regimen febrile neutropenia risk level. Associations between patient characteristics and G-CSF use were evaluated. Patients with cancer, including breast, lung, colorectal, esophageal and gastric, pancreatic, prostate, ovarian, and non-Hodgkin lymphomas, initiating myelosuppressive chemotherapy courses were included from the 2014 to 2019 commercial insurance and 2014 to 2018 Medicare fee-for-service claims databases. Data were analyzed from March to June 2021.

**Exposures:**

Year of chemotherapy initiation.

**Main Outcomes and Measures:**

The main outcomes were use and trends of G-CSFs for primary prophylaxis, from completion to 3 days after in the first chemotherapy cycle.

**Results:**

In total, 86 731 chemotherapy courses (mean [SD] age, 57.7 [11.5] years; 57 838 [66.7%] women and 28 893 [33.3%] men) were identified from 82 410 patients in the commercial insurance database and 32 398 chemotherapy courses (mean [SD] age, 71.8 [8.3] years; 18 468 [57.0%] women and 13 930 [43.0%] men) were identified from 30 279 patients in the Medicare database. Among the commercially insured population, 39 639 patients (45.7%) received G-CSFs, and 12 562 patients (38.8%) received G-CSFs among Medicare insured patients. Overall G-CSF use increased significantly throughout the study period in both populations, from 45.1% (95% CI, 44.4%-45.7%) of patients in 2014 to 47.5% (95% CI, 46.5%-48.5%) of patients in 2019 *(P* *=* .001) in the commercially insured population and from 36.0% (95% CI, 34.2%-38.0%) of patients in 2014 to 39.1% (95% CI, 38.1%-40.1%) of patients in 2018 (*P* < .001) in the Medicare population. The greatest increases in G-CSF use were observed among patients with high FN risk, from 75.0% (95% CI, 74.1%-76.0%) of patients to 83.2% (95% CI, 82.0%-84.2%) of patients (*P* < .001) among the commercially insured population and 75.3% (95% CI, 71.8%-78.6%) of patients to 86.2% (95% CI, 84.7%-87.6%) of patients (*P* < .001) among the Medicare population. Use of G-CSFs decreased in the commercially insured population among patients with intermediate FN risk (from 27.5% [95% CI, 26.4%-28.5%] of patients to 20.4% [95% CI, 19.1%-21.7%] of patients; *P* < .001) or low FN risk (from 19.3% [95% CI, 18.3%-20.4%] of patients to 16.3% [95% CI, 14.7%-18.0%] of patients; *P* < .001) and remained stable in the Medicare population (intermediate risk: from 26.4% [95% CI, 23.8%-29.2%] of patients to 28.4% [95% CI, 27.0%-29.8%] of patients; *P* = .35; low risk: from 19.6% [95% CI, 17.0%-22.4%] of patients to 20.9% [95% CI, 19.6%-22.3%] of patients; *P* = .58). Factors associated with increased odds of G-CSF use included older age (commercial insurance: adjusted odds ratio [aOR], 1.50 [95% CI, 1.41-1.59]; Medicare: aOR, 1.36 [95% CI, 1.08-1.71]), receiving a regimen with high FN risk (commercial insurance: aOR, 16.01 [95% CI, 15.17-16.90]; Medicare: aOR, 17.17 [95% CI, 15.76-18.71]), and history of neutropenia (commercial insurance: 3.90 (3.67-4.15); Medicare: 3.82 (3.50-4.18).

**Conclusions and Relevance:**

This cross-sectional study found that utilization of G-CSFs increased among patients with cancer with high FN risk in both a commercially and Medicare-insured population, but 14% to 17% of patients still did not receive preventive treatment.

## Key Points

**Question:**

What were the utilization trends associated with granulocyte colony-stimulating factors for primary prophylaxis of febrile neutropenia (FN) among patients with cancer receiving myelosuppressive chemotherapy from 2014 to 2019?

**Findings:**

This cross-sectional study of 119 129 chemotherapy courses found that among commercially and federally insured populations, use of granulocyte colony-stimulating factors increased among patients receiving high–FN risk regimens, from 75% to 83% for the commercially insured population and from 75% to 86% for the Medicare population.

**Meaning:**

This study found that there were increases in the use of granulocyte colony-stimulating factors among patients with high FN risk, but 14% to 17% of patients with high FN risk still did not receive preventive treatment.

## Teaser

This cross-sectional study assesses granulocyte colony-stimulating factor utilization trends among US adults with cancer overall and stratified by regimen febrile neutropenia risk level.

## Introduction

Myelosuppressive chemotherapy is used in most cancer treatments^1^ and is associated with numerous adverse effects, including neutropenia.^2^ Treatment may result in febrile neutropenia (FN), indicated by both neutropenia and fever.^3^ FN can interfere with treatment, decrease quality of life, and lead to prolonged hospitalizations or death if not properly managed.^4,5,6,7^

According to the National Comprehensive Care Network (NCCN) clinical practice guidelines,^3^ chemotherapy regimens with a high FN risk (≥20%) are recommended to receive primary prophylaxis using a granulocyte colony-stimulating factor (G-CSF). If a chemotherapy regimen is not considered high FN risk, additional risk factors are considered for those receiving intermediate FN risk (10%-20%) chemotherapy regimens, indicating the need for G-CSF use. These risk factors include prior chemotherapy or radiation therapy, persistent neutropenia, bone marrow involvement, recent surgical procedure, liver or renal dysfunction, and older age (≥65 years).^3^

Despite guidelines, poor use of G-CSF is reported, including underutilization among high–FN risk regimens^8,9^ and overutilization among low or intermediate FN risk regimens without other risk factors.^9,10,11^ These suboptimal prescribing patterns can have serious consequences, such as underutilization leading to lengthy inpatient stays associated with FN and overutilization causing inefficiency and waste. Quality improvement interventions implemented by certain institutions^12,13^ and payers^14^ have shown positive outcomes.

Little is known about the contemporary treatment patterns and changes on the national scale since the introduction of additional G-CSF treatment options. The development of a pegfilgrastim on-body-injector (OBI) in 2015^15^ and approvals of filgrastim and pegfilgrastim biosimilars in 2015 and 2018 have significantly expanded treatment options for patients. Unlike pegfilgrastim prefilled syringe, which requires administration 24 to 72 hours after chemotherapy administration, OBI is administered to patients on the same day of chemotherapy and delivers pegfilgrastim 27 hours later. Little is known about the impact and uptake of this device and approved biosimilars on G-CSF prescribing patterns and the uptake of these new products in the United States. Existing studies evaluated utilization at the prescription level rather than patient level,^16,17,18,19,20,21^ limiting insights into trends within certain patient groups, for example, those with high FN risk owing to regimen or patient factors.

The objective of this study was to describe patient characteristics and utilization trends of G-CSF products from 2014 to 2019, a period of new device and biosimilar availabilities. The study used 2 nationally representative samples of patients with cancer receiving myelosuppressive chemotherapy regimens, including a commercially-insured population and a Medicare fee-for-service population, to provide insights into utilization in privately and federally insured patients.

## Methods

This cross-sectional study was deemed exempt from review by the University of Florida institutional review board. Informed consent requirements were waived by the university institutional review board because the study was deemed minimal risk. This study followed the Strengthening the Reporting of Observational Studies in Epidemiology (STROBE) reporting guideline for cross-sectional studies.

### Study Design and Data Source

This was a retrospective, cohort-based, cross-sectional study. Study populations were drawn from 2 data sources: MarketScan (IBM) Commercial and Medicare Supplemental databases (2014-2019) and a 5% (2014-2015) and 15% (2016-2018) random national sample of the Medicare fee-for-service (Parts A, B, and D) claims database. Each database included patient-level enrollment information and health care utilization (inpatient, outpatient, and pharmacy services). The MarketScan commercial database is one of the largest convenient samples of privately insured patients with employer-based insurance. The MarketScan Medicare Supplemental database includes beneficiaries who possess supplemental insurance. The Medicare claims database covers individuals aged 65 years or older and selected individuals with disabilities or end-stage kidney disease.

### Study Population

Adult patients with cancer (age ≥18 years) who initiated at least 1 chemotherapy course between January 1, 2014, and November 31, 2019 (2018 for Medicare), were selected. Chemotherapy agents were identified by Healthcare Common Procedure Coding System (HCPCS) Level II codes (eTable 1 in the Supplement). For patients with multiple chemotherapy courses, all qualified courses that met the inclusion and exclusion criteria were included. The index date for each chemotherapy course was defined as the administration date of the first chemotherapy agent. Chemotherapy regimens were ascertained based on all chemotherapy claims within 7 days of the index date (day 1-7). Commonly used regimens were selected and further categorized into low (<10%), intermediate (10%-20%), or high (≥20%) risk for FN according to NCCN guidelines and clinical expert opinion (eTable 2 in the Supplement).

### Inclusion and Exclusion Criteria

We required patients to have 180 days of continuous enrollment before each chemotherapy course. We required a continuous 90-day period before the index date without claims for any chemotherapy administration. For each course, at least 2 cancer diagnoses at least 7 days apart within 30 days of the index date were required. Cancer types included breast, lung, colorectal, esophageal and gastric, pancreatic, prostate, ovarian, and non-Hodgkin lymphomas (eTable 3 in the Supplement). Chemotherapy courses were required to have no chemotherapy agent administered between day 8 and day 11. This excluded regimens with weekly administration schedules, for which pegfilgrastim is not recommended.^3^ The follow-up period started on day 1 of chemotherapy course initiation. It ended 3 days after chemotherapy completion of the first cycle, disenrollment, death, or the end of data availability (December 31, 2018, for Medicare and December 31, 2019, for commercial insurance), whichever came first.

The following exclusion criteria were applied to identified courses: (1) evidence of at least 2 primary solid cancers within 30 days of chemotherapy initiation, (2) evidence of acute myeloid leukemia within 30 days of the date of chemotherapy initiation, (3) evidence of autologous peripheral blood progenitor cell collection during the period beginning 30 days before the index date and ending 10 days after that, or (4) evidence of bone marrow transplantation or hematopoietic cell transplantation between 30 days prior or 10 days after the index date. Diagnosis and procedure codes for the exclusion criteria are shown in eTable 4 in the Supplement.

### G-CSF Utilization

G-CSF use included filgrastim, tbo-filgrastim, filgrastim-sndz, filgrastim-aafi, pegfilgrastim, pegfilgrastim-OBI, pegfilgrastim-jmdb, and pegfilgrastim-cbqv identified by National Drug Codes in pharmacy claims and HCPCS in medical claims. We considered G-CSF use as primary prophylaxis if administered within the first cycle of each chemotherapy course, from chemotherapy completion and up to 3 days afterward (eTable 5 in the Supplement). National Drug Codes and Current Procedural Terminology (CPT) codes distinguished between pegfilgrastim and pegfilgrastim-OBI (eTable 5 in the Supplement). Courses with more than 1 G-CSF product administered from chemotherapy completion to 3 days afterward were excluded.

### FN Risk Factors and Patient Characteristics

Other FN risk factors identified by the NCCN guidelines and previous studies were measured.^22,23,24^ These included age, sex, history of chemotherapy, radiation, surgical procedures, infection, neutropenia, renal disease, liver disease, cardiovascular diseases (including myocardial infarction, heart failure, peripheral vascular disease, and stroke), diabetes, chronic obstructive pulmonary disease, HIV or AIDS, metastasis, and metastatic cancer to bone. Prior chemotherapy was identified by HCPCS (eTable 1 in the Supplement). *International Classification of Diseases, Ninth Revision* (*ICD-9*)^25^ and *International Statistical Classification of Diseases and Related Health Problems, Tenth Revision* (*ICD-10*)^26^ procedure codes and CPT codes sourced from previous studies identified radiation therapy^27^ and surgical procedures.^28^ Diagnoses for other factors were measured from *ICD-9* and *ICD-10* diagnosis codes (eTable 6 in the Supplement).^25,26^ All FN risk factors were identified within 180 days before the start of the course except recent surgical procedure, which was identified 90 days before the start of the course. Because a continuous 90-day period without claims for any chemotherapy administration before the index date was required, history of chemotherapy was measured only from 180 days to 90 days before the index date.

### Statistical Analysis

Data were analyzed at the chemotherapy course level. Descriptive statistics, including mean and SD for continuous variables and frequency and percentage for categorical variables, were reported. Proportions of overall G-CSF use and G-CSF use stratified by regimen FN risk level were reported. Proportions described specific G-CSF products used among patients receiving G-CSFs. Patient characteristics were compared between patients with and without G-CSF use using *t* tests and χ^2^ tests.

Cochran-Armitage trend tests tested annual overall and stratified G-CSF utilization trends and generalized estimating equations adjusted for all examined variables and within-person correlation. All tests were 2-sided and considered significant at an α level of .05. Sensitivity analyses were performed in the subpopulations of commercially insured patients (aged <65 years) and Medicare patients (aged ≥65 years). Analyses were performed using SAS statistical software version 9.4 (SAS Institute) between March and June 2021.

## Results

### Study Populations and Characteristics

A total of 86 731 chemotherapy courses (mean [SD] age, 57.7 [11.5] years; 57 838 [66.7%] women and 28 893 [33.3%] men) were identified from 82 410 patients in the commercial insurance database, and 32 398 chemotherapy courses from 30 279 patients (mean [SD] age, 71.8 [8.3] years; 18 468 [57.0%] women and 13 930 [43.0%] men) were identified from the Medicare database. Among identified courses from commercially insured populations, 35 727 courses (41.2%) were for breast cancer, 15 210 courses (17.5%) were for lung cancer, and 12 693 courses (14.6%) were for non-Hodgkin lymphoma. Among identified courses from the Medicare population, 6383 courses (19.7%) were for breast cancer, 10 142 courses (31.3%) were for lung cancer, and 7647 courses (23.6%) were for non-Hodgkin lymphoma. The incidences of each cancer in the United States overall and in both populations during the study period are provided in eTable 7 in the Supplement.

In the commercially insured population, 37 260 patients (43.0%) received high–FN risk, 29 737 patients (34.3%) received intermediate–FN risk, and 19 734 patients (22.8%) received low–FN risk chemotherapy regimens. In the Medicare population, 7564 patients (23.4%) received high–FN risk, 13 417 patients (41.4%) received intermediate–FN risk, and 11 417 patients (35.2%) received low–FN risk chemotherapy regimens (Table 1). In the commercially insured population, 9064 patients (10.5%) had a history of neutropenia and 28 298 patients (32.6%) had a history of infection. In the Medicare population, 6955 patients (21.5%) had a history of neutropenia and 17 099 patients (52.8%) had a history of infection.

**Table 1.** Patient Characteristics by Use of G-CSF in Patients With Cancer in Commercial Insurance and Medicare Fee-for-Service Databases

Characteristic	G-CSF use, No. (%)			
	Commercial insurance (2014-2019), (n = 86 731)		Medicare (2014-2018), (n = 32 398)	
	Yes (n = 39 639)	No (n = 47 092)	Yes (n = 12 562)	No (n = 19 836)
G-CSF type				
Long-acting	38 635 (97.5)	NA	12 193 (97.1)	NA
Short-acting	1004 (2.5)	NA	369 (2.9)	NA
Age, y				
Mean (SD)	56.4 (11.7)	58.8 (11.3)	71.9 (8.5)	71.7 (7.9)
<50	10 704 (18.7)	8804 (27.0)	202 (1.6)	315 (1.6)
50-64	20 885 (52.7)	26 498 (56.3)	1115 (8.9)	2200 (11.1)
65-74	5335 (13.5)	7294 (15.5)	6925 (55.1)	9985 (50.3)
≥75	2715 (6.9)	4496 (9.6)	4320 (34.4)	7336 (37.0)
Sex^a^				
Men	7792 (19.7)	21 101 (44.8)	4332 (34.5)	9598 (48.4)
Women	31 847 (80.3)	25 991 (55.2)	8230 (65.5)	10 238 (51.6)
Cancer type^a^				
Breast	26 189 (66.1)	9538 (20.3)	4566 (36.4)	1817 (9.2)
Lung	4512 (11.4)	10 698 (22.7)	3328 (26.5)	6814 (34.4)
NHL	5416 (13.7)	7277 (15.5)	3099 (24.7)	4548 (22.9)
Colorectal	1374 (3.5)	14 166 (30.1)	461 (3.7)	4317 (21.8)
Other	1945 (4.9)	3848 (8.2)	1108 (8.8)	2340 (11.8)
Regimen FN risk level^a^				
High	28 887 (72.9)	8373 (17.8)	6275 (83.0)	1289 (6.5)
Intermediate	7200 (18.2)	22 537 (47.9)	3919 (29.2)	9498 (47.9)
Low	3552 (9.0)	16 182 (34.4)	2368 (20.7)	9049 (45.6)
Risk factors for FN				
History of chemotherapy^a^	1073 (2.7)	4624 (9.8)	666 (5.3)	2697 (13.6)
History of radiation therapy^a^	2650 (6.7)	8676 (18.4)	1363 (10.9)	4348 (21.9)
Recent surgical procedure^a^	18 690 (47.2)	18 315 (38.9)	5055 (40.2)	6345 (32.0)
History of infection^b^	11 428 (28.8)	16 870 (35.8)	6600 (52.5)	10 499 (52.9)
History of neutropenia^a^	6645 (16.8)	2419 (5.1)	3896 (31.0)	3059 (15.4)
Renal disease^a^	737 (1.9)	1186 (2.5)	1762 (14.0)	2513 (12.7)
Liver disease^a^	3685 (9.3)	7137 (15.2)	3342 (26.6)	5665 (28.6)
CVD^a^	4993 (12.6)	8540 (18.1)	5312 (42.3)	8896 (44.9)
Diabetes^a^	6502 (16.4)	8947 (19.0)	5251 (41.8)	7922 (39.9)
COPD^a^	2816 (7.1)	5109 (10.9)	4906 (39.1)	8316 (41.9)
HIV/AIDS^a^	104 (0.3)	212 (0.5)	1435 (11.4)	2000 (10.1)
Metastasis^a^	19 316 (48.7)	27 803 (59.0)	7854 (62.5)	13 369 (67.4)
Metastatic cancer to bone^b^	2522 (6.4)	5485 (11.7)	2979 (23.7)	4760 (24.0)

Abbreviations: COPD, chronic obstructive pulmonary diseases; CVD, cardiovascular disease (including myocardial infarction, heart failure, peripheral vascular disease, or stroke); FN, febrile neutropenia; G-CSF, granulocyte colony-stimulating factor; NA, not applicable; NHL, non-Hodgkin lymphoma.

^a^ Denotes *P* < .001 for the comparison of G-CSF use vs no use groups in the commercially insured and Medicare samples.

^b^ Denotes *P* < .001 for the comparison of G-CSF use vs no use groups only in the commercially insured sample.

### G-CSF Utilization

Overall, 39 639 of 86 731 patients (45.7%) in the commercially-insured population and 12 562 of 32 398 patients (38.8%) in the Medicare population used G-CSFs. In both populations, several patient characteristics differed among those with G-CSF use compared with those without (Table 1). Commercially insured patients who received G-CSF were younger than those that did not receive G-CSF (mean [SD] age, 56.4 [11.7] years vs 58.8 [11.3] years; *P* < .001), whereas the opposite was observed in the Medicare population (mean [SD] age, 71.9 [8.5] years vs 71.7 [7.9] years; *P* = .01).

In both populations, there was a higher proportion of patients receiving breast cancer chemotherapy among those receiving G-CSF than those not receiving G-CSF (commercially insured: 26 189 patients [66.1%] vs 9538 patients [20.3%]; Medicare: 4566 patients [36.4%] vs 1817 patients [9.2%]). Those who received G-CSF were more likely to receive high–FN risk regimens (commercially insured: 28 887 patients [72.9%] vs 8373 patients [17.8%]; Medicare: 6275 patients [83.0%] vs 1289 patients [6.2%]), with recent surgical procedures (commercially insured: 18 690 patients [47.2%] vs 18 315 patients [38.9%]; Medicare: 5055 patients [40.2%] vs 6345 patients [32.0%]), and neutropenia history (commercially insured: 6645 patients [16.8%] vs 2419 patients [5.1%]; Medicare: 3896 patients [31.0%] vs 3059 patients [15.4%]) . Long-acting G-CSFs accounted for nearly all G-CSF use in both populations (Table 1).

### Temporal Trends of G-CSF Utilization

**Figure 1.**
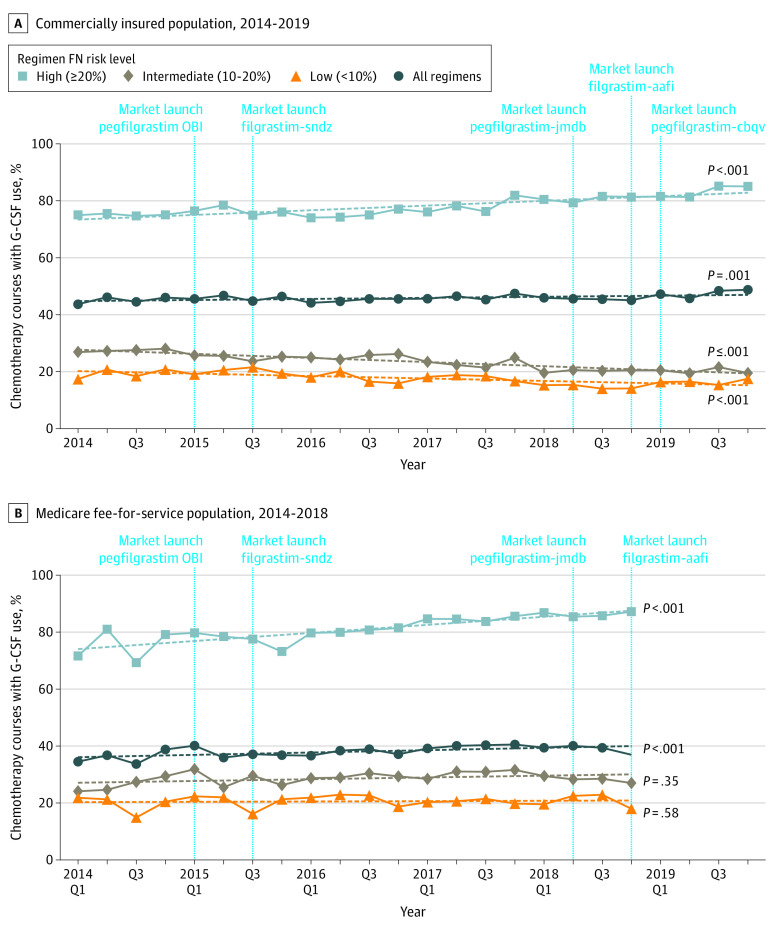
Temporal Trends of Granulocyte Colony-Stimulating Factor (G-CSF) Use FN indicates febrile neutropenia; Q, quarter.

Overall, G-CSF use increased throughout the study period in both populations. Among the commercially insured population, use increased from 45.1% (95% CI, 44.4%-45.7%) of patients in 2014 to 47.5% (95% CI, 46.5%-48.5%) of patients in 2019 (*P* = .001) (Figure 1A). The adjusted odds of G-CSF use was not significantly increased in 2019 relative to 2014. Among the Medicare population, use increased from 36.0% (95% CI 34.2%-38.0%) of patients in 2014 to 39.1% (95% CI 38.1%-40.1%) of patients in 2018 (*P* < .001) (Figure 1B), with an adjusted odds of G-CSF use of 14% (adjusted odds ratio [aOR], 1.14; 95% CI, 1.02-1.28) in 2018 relative to 2014.

The largest increases occurred among patients receiving high–FN risk regimens. In commercially insured patients, observed trends increased from 75.0% (95% CI, 74.1%-76.0%) of patients to 83.2% (95% CI, 82.0%-84.2%) of patients (*P* < .001; aOR, 1.59 [95% CI, 1.44-1.74]) (Figure 1A). Among Medicare patients, observed trends increased from 75.3% (95% CI, 71.8%-78.6%) of patients to 86.2% (95% CI 84.7%-87.6%) of patients (*P* < .001; aOR, 1.97 [95% CI, 1.58-2.47]) (Figure 1B). G-CSF use decreased among patients receiving intermediate–FN risk regimens, from 27.5% (95% CI, 26.4%-28.5%) of patients in 2014 to 20.4% (95% CI, 19.1%-21.7%) of patients in 2019 (*P* for trend < .001) and low–FN risk regimens, from 19.3% (95% CI, 18.2%-20.4%) of patients in 2014 to 16.3% (95% CI, 14.7%-18.0%) of patients in 2019 (*P* for trend < .001) in the commercially insured population (Figure 1A). G-CSF use remained stable among Medicare patients receiving intermediate–FN risk (from 26.4% [95% CI, 23.8%-29.2%] of patients in 2014 to 28.4% [95% CI, 27.0%-29.8%] of patients in 2018; *P* for trend = .35) and low–FN risk (from 19.6% [95% CI, 17.0%-22.4%] of patients in 2014 to 20.9% [95% CI, 19.6%-22.3%] of patients in 2018; *P* for trend = .58) regimens (Figure 1B).

**Figure 2.**
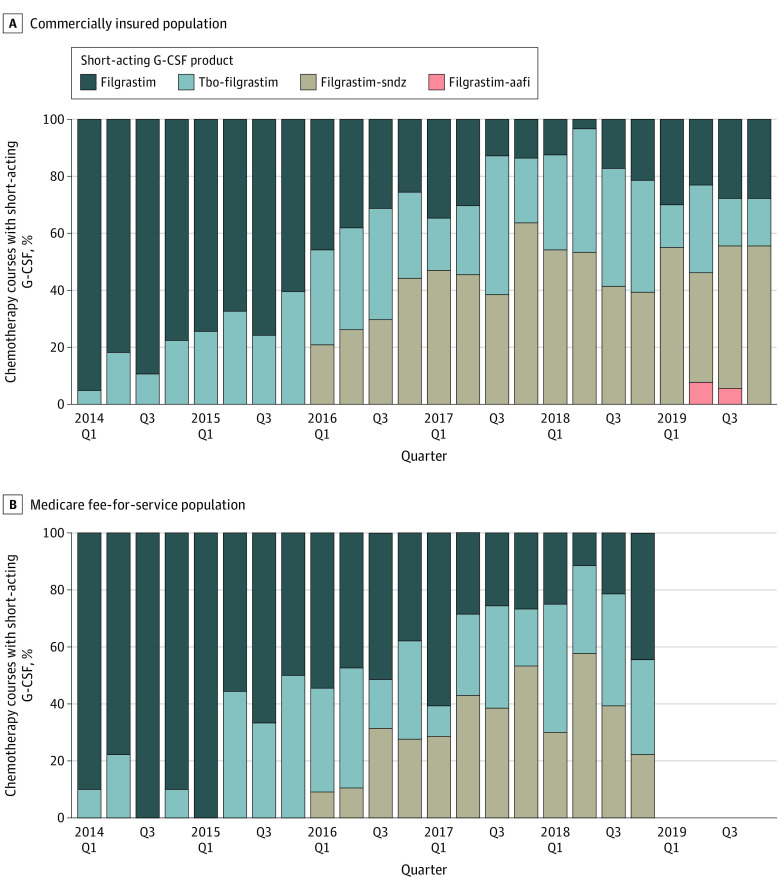
Product Choice Among All Patients Receiving Short-Acting Granulocyte Colony-Stimulating Factor (G-CSF) Q indicates quarter.

**Figure 3.**
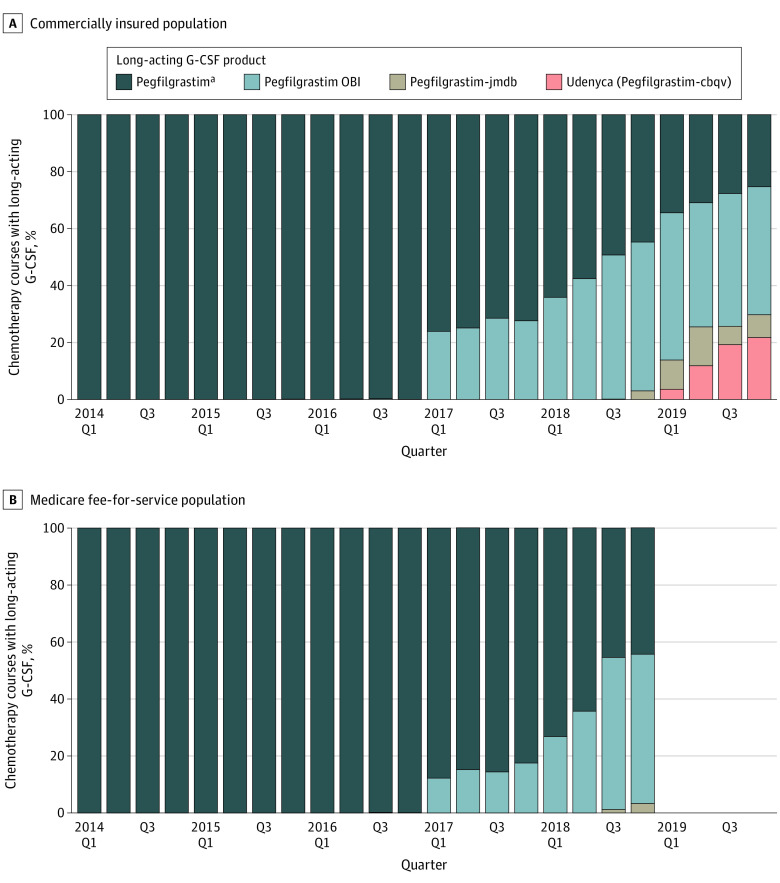
Product Choice Among All Patients Receiving Long-Acting Granulocyte Colony-Stimulating Factor (G-CSF) Pegfilgrastim includes pegfilgrastim prefilled syringe and unknown route. Q indicates quarter.

The adoption of the filgrastim biosimilar, filgrastim-sndz, was substantial in both populations, accounting for 55.6% (95% CI, 30.8%-78.5%) of all short-acting G-CSF use in the fourth quarter of 2019 in the commercially insured population (Figure 2A) and 22.2% (95% CI, 2.8%-60.0%) of all short-acting G-CSF use in the fourth quarter of 2018 in the Medicare population (Figure 2B). Among all long-acting G-CSF use in the last observable quarter of each data source, pegfilgrastim-OBI accounted for 44.9% (95% CI, 41.6%-48.3%) of use in commercial insurance data (Figure 3A) and 52.4% (95% CI, 48.5%-56.2%) of use in Medicare data (Figure 3B). The initial uptake of pegfilgrastim biosimilars was rapid, with both biosimilars accounting for a total 29.8% (95% CI, 26.8%-32.9%) of all long-acting G-CSF use in the fourth quarter of 2019 in the commercially insured population.

### Factors Associated With G-CSF Use

Older age was significantly associated with use of G-CSF in both populations (aged 65-74 years vs aged <50 years: commercially insured: aOR, 1.50; 95% CI, 1.41-1.59; Medicare: aOR, 1.36; 95% CI, 1.08-1.71). While older age was significantly associated with higher odds of receiving G-CSF in almost all commercial insurance subpopulations stratified by FN risk level, it was significantly associated with G-CSF use in some, but not all, Medicare subpopulations stratified by FN risk level (Table 2).

**Table 2.** Year of Chemotherapy Initiation and Patient Characteristics Associated With Use of Granulocyte Colony-Stimulating Factor by Regimen Febrile Neutropenia Risk Category

Characteristic	Regimen FN risk level, adjusted odds ratio (95% CI)^a^							
	Commercial insurance				Medicare			
	All (n = 86 731)	High (n = 37 260)	Intermediate (n = 29 737)	Low (n = 19 734	All (n = 32 398)	High (n = 7564)	Intermediate (n = 13 417)	Low (n = 11 417)
Year of chemotherapy initiation								
2014	1 [Reference]	1 [Reference]	1 [Reference]	1 [Reference]	1 [Reference]	1 [Reference]	1 [Reference]	1 [Reference]
2015	0.97 (0.93-1.03)	1.06 (0.98-1.14)	0.85 (0.78-0.92)^b^	1.06 (0.95-1.18)	1.11 (0.96-1.28)	1.11 (0.84-1.46)	1.12 (0.91-1.37)	1.11 (0.86-1.42)
2016	0.87 (0.83-0.92)^b^	0.93 (0.86-1.01)	0.80 (0.73-0.88)^b^	0.86 (0.76-0.97)^b^	1.12 (1.00-1.26)	1.37 (1.09-1.72)^b^	1.03 (0.87-1.22)	1.05 (0.85-1.28)
2017	0.92 (0.87-0.97)^b^	1.09 (1.01-1.18)^b^	0.73 (0.67-0.80)^b^	0.89 (0.78-1.01)	1.17 (1.04-1.31)^b^	1.76 (1.41-2.20)^b^	1.04 (0.88-1.24)	1.01 (0.83-1.22)
2018	0.92 (0.87-0.97)^b^	1.30 (1.20-1.42)^b^	0.65 (0.58-0.71)^b^	0.73 (0.64-0.83)^b^	1.14 (1.02-1.28)^b^	1.97 (1.58-2.47)^b^	0.96 (0.81-1.14)	1.00 (0.82-1.22)
2019	1.02 (0.96-1.08)	1.59 (1.44-1.74)^b^	0.64 (0.58-0.72)^b^	0.86 (0.74-0.99)^b^	NA	NA	NA	NA
Age, y								
<50	1 [Reference]	1 [Reference]	1 [Reference]	1 [Reference]	1 [Reference]	1 [Reference]	1 [Reference]	1 [Reference]
50-64	1.06 (1.02-1.11)^b^	1.01 (0.95-1.06)	1.30 (1.19-1.42)^b^	1.05 (0.92-1.20)	0.98 (0.77-1.24)	0.78 (0.52-1.18)	0.98 (0.68-1.42)	1.42 (0.82-2.45)
65-74	1.50 (1.41-1.59)^b^	1.37 (1.24-1.51)^b^	1.77 (1.60-1.96)^b^	1.46 (1.26-1.68)^b^	1.31 (1.04-1.64)^b^	1.33 (0.91-1.96)	1.25 (0.88-1.78)	1.74 (1.03-2.94)
≥75	1.50 (1.41-1.59)^b^	1.67 (1.43-1.95)^b^	1.77 (1.60-1.96)^b^	1.45 (1.24-1.70)^b^	1.36 (1.08-1.71)^b^	1.27 (0.86-1.89)	1.34 (0.94-1.92)	1.88 (1.11-3.18)^b^
Sex (women vs men)	1.27 (1.22-1.32)^b^	1.61 (1.48-1.75)^b^	1.15 (1.09-1.21)^b^	1.13 (1.05-1.22)^b^	1.03 (0.97-1.09)	1.20 (1.02-1.41)^b^	1.00 (0.92-1.08)	1.00 (0.91-1.10)
Regimen FN risk level								
High	16.01 (15.17-16.90)^b^	NA	NA	NA	17.17 (15.76-18.71)^b^	NA	NA	NA
Intermediate	1.55 (1.48-1.62)^b^	NA	NA	NA	1.56 (1.46-1.66)^b^	NA	NA	NA
Low	1 [Reference]	NA	NA	NA	1 [Reference]	NA	NA	NA
Risk factors for FN								
History of chemotherapy	0.53 (0.49-0.58)^b^	0.36 (0.28-0.45)^b^	0.85 (0.76-0.95)^b^	0.38 (0.33-0.43)^b^	0.48 (0.43-0.53)^b^	0.49 (0.36-0.66)^b^	0.83 (0.71-0.98)^b^	0.30 (0.26-0.36)^b^
History of radiation therapy	0.72 (0.68-0.76)^b^	0.79 (0.69-0.91)^b^	0.66 (0.61-0.71)^b^	0.84 (0.76-0.94)^b^	0.62 (0.57-0.67)^b^	0.51 (0.40-0.66)^b^	0.56 (0.51-0.62)^b^	0.84 (0.73-0.97)^b^
Recent surgical procedure	0.87 (0.84-0.90)^b^	0.93 (0.89-0.98)^b^	0.65 (0.61-0.69)^b^	1.26 (1.16-1.38)^b^	0.96 (0.91-1.02)	1.10 (0.97-1.26)	0.74 (0.68-0.81)^b^	1.33 (1.19-1.48)^b^
History of infection	1.02 (0.98-1.05)	0.98 (0.93-1.04)	1.05 (0.99-1.11)	1.06 (0.98-1.14)	1.09 (1.03-1.16)^b^	1.02 (0.88-1.17)	1.16 (1.07-1.26)^b^	1.07 (0.96-1.18)
History of neutropenia	3.90 (3.67-4.15)^b^	3.13^b^ (2.85-3.44)	4.81 (4.35-5.32)^b^	3.98 (3.55-4.46)^b^	3.82 (3.50-4.18)^b^	2.17 (1.81-2.60)^b^	4.38 (3.84-4.99)^b^	4.34 (3.77-5.00)^b^
Kidney disease	0.98 (0.87-1.10)	1.02 (0.82-1.28)	0.89 (0.75-1.05)	0.99 (0.80-1.23)	0.87 (0.74-1.03)	0.83 (0.60-1.15)	1.07 (0.85-1.34)	0.81 (0.59-1.11)
Liver disease	0.92 (0.88-0.97)^b^	1.04 (0.94-1.14)	0.95 (0.88-1.03)	0.94 (0.84-1.06)	0.88 (0.82-0.95)^b^	0.92 (0.76-1.10)	0.91 (0.82-1.00)	0.94 (0.83-1.08)
CVD	1.06 (1.01-1.12)^b^	1.01 (0.92-1.11)	1.13 (1.05-1.21)^b^	1.01 (0.92-1.11)	0.99 (0.93-1.05)	0.95 (0.82-1.11)	1.01 (0.93-1.10)	0.97 (0.88-1.08)
Diabetes	1.05 (1.00-1.09)^b^	1.03 (0.96-1.11)	1.06 (0.99-1.13)	1.04 (0.95-1.15)	0.98 (0.92-1.04)	0.90 (0.78-1.03)	1.03 (0.94-1.12)	0.97 (0.87-1.08)
COPD	1.34 (1.26-1.42)^b^	1.09 (0.95-1.26)	1.63 (1.50-1.77)^b^	1.17 (1.05-1.31)^b^	1.25 (1.17-1.33)^b^	0.86 (0.73-1.02)	1.48 (1.36-1.62)^b^	1.17 (1.05-1.30)^b^
HIV/AIDS	0.79 (0.59-1.05)	0.96 (0.60-1.54)	0.50 (0.31-0.83)^b^	1.52 (0.81-2.87)	0.21 (0.17-0.26)^b^	1.18 (0.78-1.80)	0.10 (0.07-0.14)^b^	0.25 (0.16-0.40)^b^
Metastasis	1.04 (1.00-1.07)^b^	1.22 (1.16-1.29)^b^	0.86 (0.81-0.91)^b^	0.81 (0.74-0.88)^b^	0.94 (0.89-1.01)	1.02 (0.88-1.18)	0.90 (0.82-0.99)^b^	0.85 (0.76-0.95)^b^
Metastatic cancer to bone	1.26 (1.18-1.34)^b^	0.64 (0.54-0.76)^b^	1.44 (1.33-1.56)^b^	1.24 (1.10-1.40)^b^	1.41 (1.30-1.53)^b^	0.65 (0.49-0.85)^b^	1.56 (1.40-1.73)^b^	1.24 (1.06-1.45)^b^

Abbreviations: COPD, chronic obstructive pulmonary diseases; CVD, cardiovascular disease (includes myocardial infarction, heart failure, peripheral vascular disease, or stroke); FN, febrile neutropenia; NA, not applicable.

^a^ Adjusted odds ratios were derived from multivariable logistic regression controlling for year, age, sex, cancer type, regimen FN risk level (only in models for all regimens), history of chemotherapy, history of radiation therapy, recent surgery, history of infection, history of neutropenia, and presence of kidney disease, liver disease, cardiovascular diseases, diabetes, chronic obstructive pulmonary disease, HIV/AIDS, metastasis, and metastatic cancer to bone.

^b^ Denotes statistically significant results at *P* < .05.

Regimen FN risk level was significantly associated with G-CSF use in both populations. Patients receiving regimens with high FN risk were 16-fold more likely to use G-CSFs (aOR, 16.01; 95% CI 15.17-16.90) in the commercially insured population and 17-fold more likely to use G-CSFs (aOR, 17.17; 95% CI 15.76-18.71) in the Medicare population compared with patients receiving low–FN risk regimens. Patients receiving intermediate–FN risk regimens were 55% more likely to receive G-CSFs (aOR, 1.55; 95% CI 1.48-1.62) than those receiving low–FN risk regimens in the commercially insured population and 56% more likely to use G-CSFs (aOR, 1.56; 95% CI 1.46-1.66) in the Medicare population (Table 2).

Some FN risk factors were inversely associated with G-CSF use (eg, chemotherapy and radiation history), and some FN risk factors were associated with increased odds of G-CSF use (eg, history of neutropenia, liver disease, chronic obstructive pulmonary disease, and metastatic cancer to bone). Compared with patients without a history of neutropenia, patients with previous neutropenia had 3.9-fold higher odds of using G-CSFs (aOR, 3.90; 95% CI 3.62-4.15) among the commercially-insured population and 3.8-fold higher odds of using G-CSFs (aOR, 3.82; 95% CI 3.50-4.18) in the Medicare population (Table 2). Analyses restricting to patients younger than 65 years in the commercially insured population and aged 65 years or older in the Medicare population were consistent with the primary analyses (eTable 8 and eTable 9 in the Supplement).

## Discussion

This cross-sectional study is the first population-based study, to our knowledge, to describe G-CSF use in 2 nationally representative databases and the first regimen- and patient-level analysis reporting the uptake of both filgrastim and pegfilgrastim biosimilars among patients with cancer receiving myelosuppressive chemotherapy stratified by risk of FN. While prior studies used data prior to 2011 and reported underutilization of G-CSF among patients with cancer receiving high–FN risk regimens,^8,29^ our study showed a gradual increase in G-CSF use among patients receiving high–FN risk regimens in the past 5 years in both populations.

These findings indicate possible improvements in G-CSF use among patients with high FN risk in clinical practice. However, 14% to 17% of patients with high FN risk still did not receive prophylactic G-CSF in the last observable quarter in both populations. According to NCCN guidelines,^3^ the appropriateness of G-CSF use among patients receiving intermediate– or low–FN risk regimens is dependent on other risk factors and clinical judgment. Drivers of the observed decreases in G-CSF use among patients receiving intermediate– or low–FN risk regimens in the commercially insured population may be related to implementing practice improvement policy initiatives^12^ and decision support tools^14,30^ aimed to reduce overutilization and inefficient practices in such patients. Despite observed reductions, the appropriateness of continued G-CSF use among patients with intermediate or low risk should be determined to promote efficient use of G-CSFs.

The availability of biosimilar G-CSF presents an opportunity to reassess the cost-effectiveness of prophylactic G-CSF use in patients receiving myelosuppressive chemotherapy regimens. Evidence shows that using biosimilar G-CSFs for primary prophylaxis among patients with intermediate risk can be a cost-effective strategy even without risk factors.^31,32,33^ Moreover, short-term guidance issued by NCCN also states that prophylactic G-CSF use among patients receiving intermediate-risk regimens may be considered in the context of the COVID-19 pandemic.^34^ New evidence and guidelines highlight the need for future studies focusing on this patient subgroup to inform clinical and reimbursement decision-making.

G-CSF product choice in this study was consistent with current evidence^22^ and prior prescription-level utilization studies.^16,17,18,19,20^ Use of the filgrastim biosimilar, filgrastim-sndz, surpassed that of the filgrastim originator, and pegfilgrastim biosimilars were also rapidly adopted. However, while we observed the adoption of G-CSF biosimilars, especially filgrastim, the market is dominated by long-acting G-CSF products, which is itself dominated by the branded OBI delivery device (44.9%-52.4% of all long-acting G-CSF use).

Preference for the OBI device may limit the adoption of long-acting biosimilar G-CSF products and undermine the potential cost savings implied with biosimilar availability in this therapeutic area. While a simulation study has demonstrated cost-savings from biosimilar adoption,^35^ a variety of barriers exist.^36,37,38^ It may not be cost-saving for patients considering the need for a second visit and associated costs. Similar device development or more aggressive reimbursement or formulary structures may be needed to drive the utilization of biosimilar G-CSF products. Future studies demonstrating cost-savings from pegfilgrastim biosimilars using real-world data may help strengthen payer and clinician confidence and facilitate broader adoption.

Among patients receiving high–FN risk regimens, few FN risk factors were associated with the use of G-CSF. This suggests other factors not captured in this study, such as race, rurality, plan type, region, clinician type, physician training, prescribing preferences, and patient preferences, may influence G-CSF use. Future studies are needed to understand further the complexity of these prescribing choices and associated factors, particularly to evaluate risk factors and regimen risk within individual cancer types.

### Limitations

Our study has several limitations. Although we quantified the FN risk level for selected chemotherapy regimens, the possibility of misclassification, either overestimation or underestimation of FN risk, cannot be ruled out. Second, we excluded regimens with weekly schedules, which are common in patients with breast cancer. While this approach is used widely in the literature,^22,39^ some regimens may be excluded and limits generalizability to evaluated regimens. Third, the identification of pegfilgrastim-OBI was primarily based on a combination of HCPCS and CPT codes, which may miss some pegfilgrastim-OBI if codes are incorrectly submitted. Fourth, results are only generalizable to the populations covered by each database. The incidence of cancer estimated for the United States overall and each database shown in eTable 7 in the Supplement provides some basis for better understand the generalizability of the study findings.

## Conclusions

In two nationally representative samples, there was increased G-CSF use among patients with high FN risk from 2014 to 2019 in a commercially insured population and from 2014 to 2018 in the Medicare population, but 14% to 17% of patients with high risk did not receive prophylaxis. Older age, receiving a high–FN risk regimen, and history of neutropenia were associated with use of G-CSF.

## Notes

Supplement. **eTable 1.** HCPCS for Chemotherapy **eTable 2.** Chemotherapy Regimen FN Risk Level **eTable 3.** *ICD-9* and *ICD-10* Diagnosis Codes for Cancer **eTable 4.** Diagnosis and Procedure Codes for Exclusion Criteria **eTable 5.** NDC, HCPCS, and CPT Codes for G-CSF Product **eTable 6.** *ICD-9* and *ICD-10* Diagnosis Codes for FN Risk Factors **eTable 7.** Cancer Incidence by Site in Overall US Population, Commercial Insurance, and Medicare FFS Database **eTable 8.** Year of Chemotherapy Initiation and Patient Characteristics Associated with Use of G-CSF by Regimen FN Risk Category Among Patients Younger Than 65 Years in the Commercially Insured Population **eTable 9.** Year of Chemotherapy Initiation and Patient Characteristics Associated with Use of G-CSF by Regimen FN Risk Category Among Patients Aged 65 Years or Older in Medicare Population
