# Supplementary material for: Trends in Use of Granulocyte Colony-Stimulating Factor Following Introduction of Biosimilars Among Adults With Cancer and Commercial or Medicare Insurance From 2014 to 2019
Source: JAMA Netw Open. 2021 Nov 23;4(11):e2133474. doi: 10.1001/jamanetworkopen.2021.33474 (PMC8611485; doi:10.1001/jamanetworkopen.2021.33474)
Supplement: Supplement. — eTable 1. HCPCS for Chemotherapy eTable 2. Chemotherapy Regimen FN Risk Level eTable 3. ICD-9 and ICD-10 Diagnosis Codes for Cancer eTable 4. Diagnosis and Procedure Codes for Exclusion Criteria eTable 5. NDC, HCPCS, and CPT Codes for G-CSF Product eTable 6. ICD-9 and ICD-10 Diagnosis Codes for FN Risk Factors eTable 7. Cancer Incidence by Site in Overall US Population, Commercial Insurance, and Medicare FFS Database eTable 8. Year of Chemotherapy Initiation and Patient Characteristics Associated with Use of G-CSF by Regimen FN Risk Category Among Patients Younger Than 65 Years in the Commercially Insured Population eTable 9. Year of Chemotherapy Initiation and Patient Characteristics Associated with Use of G-CSF by Regimen FN Risk Category Among Patients Aged 65 Years or Older in Medicare Population [file jamanetwopen-e2133474-s001.pdf]

Supplemental Online Content

Wang CY, Heldermon CD, Vouri SM, et al. Trends in use of granulocyte colony-stimulating factor following introduction of biosimilars among adults with cancer and commercial or Medicare insurance from 2014 to 2019. *JAMA Netw Open*. 2021;4(11):e2133474. doi:10.1001/jamanetworkopen.2021.33474

eTable 1. HCPCS for Chemotherapy
eTable 2. Chemotherapy Regimen FN Risk Level
eTable 3. *ICD-9* and *ICD-10* Diagnosis Codes for Cancer
eTable 4. Diagnosis and Procedure Codes for Exclusion Criteria
eTable 5. NDC, HCPCS, and CPT Codes for G-CSF Product
eTable 6. *ICD-9* and *ICD-10* Diagnosis Codes for FN Risk Factors
eTable 7. Cancer Incidence by Site in Overall US Population, Commercial Insurance, and Medicare FFS Database
eTable 8. Year of Chemotherapy Initiation and Patient Characteristics Associated with Use of G-CSF by Regimen FN Risk Category Among Patients Younger Than 65 Years in the Commercially Insured Population
eTable 9. Year of Chemotherapy Initiation and Patient Characteristics Associated with Use of G-CSF by Regimen FN Risk Category Among Patients Aged 65 Years or Older in Medicare Population

This supplemental material has been provided by the authors to give readers additional information about their work.

© 2021 Wang CY et al. *JAMA Network Open.*

eTable 1. HCPCS for Chemotherapy

Agent	HCPCS	Agent	HCPCS
Bendamustine	J9033, J9034	Daunorubicin	J9151, J9150
Busulfan	J0594, J8510	Doxorubicin	J9000, J9002, Q2048, Q2049, Q2050
Carboplatin	J9045	Epirubicin	J9178
Carmustine	J9050	Idarubicin	J9211
Chlorambucil	S0172	ixabepilone	C9240, J9207
Cisplatin	J9060	Mitomycin	J9280
Cyclophosphamide	J9070, J9092, J9094, J9097, J8530	Mitoxantrone	J9293
Dacarbazine	J9130	Plicamycin	J9270
Ifosfamide	J9208	Valrubicin	J9357
Lomustine	S0178	Avelumab	C9491
Mechlorethamine	J9230	Atezolizumab	C9483
Melphalan	J9245, J8600	Durvalumab	C9492
Oxaliplatin	J9263	Ipilimumab	J9228
Procarbazine	S0182	Nivolumab	J9299, C9453
Streptozocin	J9320	Pembrolizumab	C9027, J9271
Temozolomide	J8700, J9328, C9253, C1086	Ado-Trastuzumab Emtansine	J9354, C9131
Thiotepa	J9340	Brentuximab vedotin	J9042
Trabectedin	C9480	Gemtuzumab ozogamicin	J9300, J9203
Azacitidine	J9025	Alemtuzumab	J0202, Q9979, J9010
Capecitabine	J8520, J8521	Bevacizumab	C9257, J9035
Cladribine	J9065	Blinatumomab	C9449, J9039
Clofarabine	J9027	Cetuximab	J9055
Cytarabine	J9100, J9098	Daratumumab	J9415, C9476
Decitabine	J0894	Denosumab	J0897
Floxuridine	J9200	Elotuzumab	C9477, J9176
Fludarabine	J9185, J8562	Necitumumab	C9475, J9295
Fluorouracil	J9190	Obinutuzumab	C9021, J9301
Gemcitabine	J9201	Ofatumumab	J9302
Mercaptopurine	S0108	Olaratumab	J9285

© 2021 Wang CY et al. *JAMA Network Open.*

Agent	HCPCS	Agent	HCPCS
Methotrexate	J8610, J9250, J9260	Panitumumab	J9303
Nelarabine	J9261	Pertuzumab	C9292, J9306
Pemetrexed	J9305	Ramucirumab	C9025, J9308
Pentostatin	J9268	Rituximab	J9310
Pralatrexate	J9307	Siltuximab	C9455, J2860
Trimetrexate Glucuronate	J3305	Trastuzumab	J9355
Cabazitaxel	J9043	Etoposide	J9181, J8560
Docetaxel	J9171	Omacetaxine	C9297, J9262
Eribulin mesylate	J9179	Teniposide	Q2017
Paclitaxel	J9264, J9265, J9267	Bortezomib	J9041
Vinblastine	J9360	Carfilzomib	J9047, C9295
Vincristine	J9370, J9371	Tositumomab	A9534, A9545
Vinorelbine	J9390	Ziv-aflibercept	C9296, J9400
Bleomycin	J9040	Irinotecan	J9206, C9474
Dactinomycin	J9120	Topotecan	J8705, J9351

HCPCS= Healthcare common procedure coding system

© 2021 Wang CY et al. *JAMA Network Open.*

eTable 2. Chemotherapy Regimen FN Risk Level

FN risk level	Regimen
High	▪ Breast cancer: carboplatin + docetaxel trastuzumab (+ pertuzumab)<br>▪ Breast cancer: cyclophosphamide + docetaxel<br>▪ Breast cancer: cyclophosphamide + doxorubicin<br>▪ Breast cancer: cyclophosphamide + docetaxel + doxorubicin<br>▪ Breast cancer: cyclophosphamide + docetaxel + trastuzumab<br>▪ Breast cancer: cyclophosphamide + epirubicin + fluorouracil<br>▪ Breast cancer: doxorubicin (14-day cycle)<br>▪ Lung cancer: topotecan<br>▪ NHL: carboplatin + etoposide + ifosfamide + rituximab<br>▪ NHL: cyclophosphamide + doxorubicin + etoposide + vincristine (+ rituximab)<br>▪ NHL: cyclophosphamide + doxorubicin + methotrexate + rituximab + vincristine<br>▪ NHL: cyclophosphamide + doxorubicin + rituximab<br>▪ NHL: cyclophosphamide + doxorubicin+ vincristine (+ rituximab)<br>▪ NHL: cyclophosphamide + etoposide + rituximab + vincristine<br>▪ NHL: cyclophosphamide + fludarabine + rituximab<br>▪ NHL: gemcitabine + oxaliplatin + rituximab
Intermediate	▪ Breast cancer: carboplatin + docetaxel OR paclitaxel<br>▪ Breast cancer: cyclophosphamide + fluorouracil + methotrexate<br>▪ Breast cancer: docetaxel (+ trastuzumab) (+ pertuzumab)<br>▪ Breast cancer: paclitaxel<br>▪ Colorectal cancer: oxaliplatin + fluorouracil + bevacizumab<br>▪ Colorectal cancer: fluorouracil + mitomycin<br>▪ Colorectal cancer: fluorouracil + oxaliplatin<br>▪ Esophageal and gastric cancer: cisplatin + docetaxel + fluorouracil<br>▪ Lung cancer: carboplatin + paclitaxel + bevacizumab<br>▪ Lung cancer: carboplatin + etoposide<br>▪ Lung cancer: carboplatin + docetaxel OR paclitaxel<br>▪ Lung cancer: carboplatin + paclitaxel + pembrolizumab<br>▪ Lung cancer: cisplatin + docetaxel<br>▪ Lung cancer: cisplatin + etoposide<br>▪ Lung cancer: docetaxel (+ ramucirumab)<br>▪ NHL: cyclophosphamide + mitoxantrone + rituximab + vincristine<br>▪ NHL: cyclophosphamide + vincristine (+ rituximab)<br>▪ NHL: cytarabine

© 2021 Wang CY et al. *JAMA Network Open.*

FN risk level	▪ Regimen
Intermediate	▪ Ovarian cancer: carboplatin + docetaxel<br>▪ Pancreatic cancer: fluorouracil + irinotecan + oxaliplatin<br>▪ Prostate cancer: cabazitaxel<br>▪ Prostate cancer: docetaxel
Low	▪ Breast cancer: cyclophosphamide<br>▪ Breast cancer: eribulin mesylate<br>▪ Colorectal cancer: irinotecan + fluorouracil (+ bevacizumab)<br>▪ Colorectal cancer: irinotecan + fluorouracil (+cetuximab)<br>▪ Esophageal and gastric cancer: fluorouracil + oxaliplatin<br>▪ Esophageal and gastric cancer: fluorouracil + oxaliplatin + docetaxel<br>▪ Lung cancer: bevacizumab + carboplatin + pemetrexed<br>▪ Lung cancer: carboplatin<br>▪ Lung cancer: carboplatin + pemetrexed (+ pembrolizumab)<br>▪ Lung cancer: cisplatin + pemetrexed<br>▪ Lung cancer: etoposide<br>▪ Lung cancer: pemetrexed<br>▪ NHL: bendamustine (+ rituximab)<br>▪ NHL: cyclophosphamide + rituximab<br>▪ NHL: rituximab<br>▪ Pancreatic cancer: fluorouracil<br>▪ Pancreatic cancer: fluorouracil + irinotecan<br>▪ Pancreatic cancer: fluorouracil + oxaliplatin

FN= Febrile neutropenia; NHL= non-Hodgkin lymphoma

© 2021 Wang CY et al. *JAMA Network Open.*

eTable 3. *ICD-9* and *ICD-10* Diagnosis Codes for Cancer

**Cancer type**	**ICD-9/10 diagnosis codes**
Breast cancer	**9**: 1740, 1741, 1742, 1743, 1744, 1745, 1746, 1748, 1749, 1750, 1759<br>**10**: C50011, C50012, C50019, C50021, C50022, C50029, C50111, C50112, C50119, C50121, C50122, C50129, C50211, C50212, C50219, C50221, C50222, C50229, C50311, C50312, C50319, C50321, C50322, C50329, C50411, C50412, C50419, C50421, C50422, C50429, C50511, C50512, C50519, C50521, C50522, C50529, C50611, C50612, C50619, C50621, C50622, C50629, C50811, C50812, C50819, C50821, C50822, C50829, C50911, C50912, C50919, C50921, C50922, C50929
Lung cancer	**9**: 1622, 1623, 1624, 1625, 1628, 1629, 20921<br>**10**: C3400, C3401, C3402, C3410, C3411, C3412, C342, C3430, C3431, C3432, C3480, C3481, C3482, C3490, C3491, C3492, C7A090
Colorectal cancer	**9**: 1530, 1531, 1532, 1533, 1534, 1535, 1536, 1537, 1538, 1539, 1590, 20910, 20911, 20912, 20913, 20914, 20915, 20916, 1540, 1541, 1542, 1543, 1548, 20917, 79670, 79671, 79672, 79673, 79674, 79676<br>**10**: C180, C181, C182, C183, C184, C185, C186, C187, C188, C189, C260, C49A4, C7A020, C7A021, C7A022, C7A023, C7A024, C7A025, C7A029, C19, C20, C49A5, C210, C211, C212, C218, C7A026
Esophageal and Gastric cancer	**9**: 1500, 1501, 1502, 1503, 1504, 1505, 1508, 1509, 1510, 1511, 1512, 1513, 1514, 1515, 1516, 1518, 1519, 20923<br>**10**: C153, C154, C155, C158, C159, C49A1, C160, C161, C162, C163, C164, C165, C166, C168, C169, C49A2, C49A3, C7A092
Pancreatic cancer	**9**: 1570, 1571, 1572, 1573, 1574, 1578, 1579<br>**10**: C250, C251, C252, C253, C254, C257, C258, C259
Prostate cancer	**9**: 185<br>**10**: C61
Ovarian cancer	**9**: 1830<br>**10**: C561, C562, C569
Non-Hodgkin's lymphomas	**9**: 20000, 20001, 20002, 20003, 20004, 20005, 20006, 20007, 20008, 20010, 20011, 20012, 20013, 20014, 20015, 20016, 20017, 20018, 20020, 20021, 20022, 20023, 20024, 20025, 20026, 20027, 20028, 20030, 20031, 20032, 20033, 20034, 20035, 20036, 20037, 20038, 20040, 20041, 20042, 20043, 20044, 20045, 20046, 20047, 20048, 20050, 20051, 20052, 20053, 20054, 20055, 20056, 20057, 20058,

© 2021 Wang CY et al. *JAMA Network Open.*

Cancer type	ICD-9/10 diagnosis codes
Non-Hodgkin's lymphomas	**9**: 20060, 20061, 20062, 20063, 20064, 20065, 20066, 20067, 20068, 20070, 20071, 20072, 20073, 20074, 20075, 20076, 20077, 20078, 20080, 20081, 20082, 20083, 20084, 20085, 20086, 20087, 20088, 20200, 20201, 20202, 20203, 20204, 20205, 20206, 20207, 20208, 20210, 20211, 20212, 20213, 20214, 20215, 20216, 20217, 20218, 20220, 20221, 20222, 20223, 20224, 20225, 20226, 20227, 20228, 20270, 20271, 20272, 20273, 20274, 20275, 20276, 20277, 20278, 20280, 20281, 20282, 20283, 20284, 20285, 20286, 20287, 20288, 20290, 20291, 20292, 20293, 20294, 20295, 20296, 20297, 20298<br>**10**: C964, C8200, C8201, C8202, C8203, C8204, C8205, C8206, C8207, C8208, C8209, C8210, C8211, C8212, C8213, C8214, C8215, C8216, C8217, C8218, C8219, C8220, C8221, C8222, C8223, C8224, C8225, C8226, C8227, C8228, C8229, C8230, C8231, C8232, C8233, C8234, C8235, C8236, C8237, C8238, C8239, C8240, C8241, C8242, C8243, C8244, C8245, C8246, C8247, C8248, C8249, C8250, C8251, C8252, C8253, C8254, C8255, C8256, C8257, C8258, C8259, C8260, C8261, C8262, C8263, C8264, C8265, C8266, C8267, C8268, C8269, C8280, C8281, C8282, C8283, C8284, C8285, C8286, C8287, C8288, C8289, C8290, C8291, C8292, C8293, C8294, C8295, C8296, C8297, C8298, C8299, C8300, C8301, C8302, C8303, C8304, C8305, C8306, C8307, C8308, C8309, C8310, C8311, C8312, C8313, C8314, C8315, C8316, C8317, C8318, C8319, C8330, C8331, C8332, C8333, C8334, C8335, C8336, C8337, C8338, C8339, C8350, C8351, C8352, C8353, C8354, C8355, C8356, C8357, C8358, C8359, C8370, C8371, C8372, C8373, C8374, C8375, C8376, C8377, C8378, C8379, C8380, C8381, C8382, C8383, C8384, C8385, C8386, C8387, C8388, C8389, C8390, C8391, C8392, C8393, C8394, C8395, C8396, C8397, C8398, C8399, C8400, C8401, C8402, C8403, C8404, C8405, C8406, C8407, C8408, C8409, C8410, C8411, C8412, C8413, C8414, C8415, C8416, C8417, C8418, C8419, C8440, C8441, C8442, C8443, C8444, C8445, C8446, C8447, C8448, C8449, C8460, C8461, C8462, C8463, C8464, C8465, C8466, C8467, C8468, C8469, C8470, C8471, C8472, C8473, C8474, C8475, C8476, C8477, C8478, C8479, C8490, C8491, C8492, C8493, C8494, C8495, C8496, C8497, C8498, C8499, C84A0, C84A1, C84A2, C84A3, C84A4, C84A5, C84A6, C84A7, C84A8, C84A9, C84Z0, C84Z1, C84Z2, C84Z3, C84Z4, C84Z5, C84Z6, C84Z7, C84Z8, C84Z9, C8510, C8511, C8512, C8513, C8514, C8515, C8516, C8517, C8518, C8519,

© 2021 Wang CY et al. *JAMA Network Open.*

Cancer type	ICD-9/10 diagnosis codes
Non-Hodgkin's lymphomas	**10**: C8520, C8521, C8522, C8523, C8524, C8525, C8526, C8527, C8528, C8529, C8580, C8581, C8582, C8583, C8584, C8585, C8586, C8587, C8588, C8589, C8590, C8591, C8592, C8593, C8594, C8595, C8596, C8597, C8598, C8599, C860, C861, C862, C863, C864, C865, C866, C880, C882, C883, C884, C888, C889, C969, C96Z

© 2021 Wang CY et al. *JAMA Network Open.*

eTable 4. Diagnosis and Procedure Codes for Exclusion Criteria

Exclusion criteria	ICD-9/10 diagnosis and procedure code, HCPCS
AML	**ICD-9 diagnosis**: 205.0, 205.00, 205, 205.02, 205.2, 205.20, 205.22, 205.3, 205.30, 205.32, 205.9, 205.90, 205.92<br>**ICD-10 diagnosis**: C92, C92.0, C92.00, C92.02, C92.1, C92.10, C92.12, C92.2, C92.20, C92.22, C92.3, C92.30, C92.32, C92.4, C92.40, C92.42, C92.5, C92.50, C92.52, C92.6, C92.60, C92.62, C92.9, C92.90, C92.92, C92.A, C92.A0, C92.A2, C92.Z, C92.Z0, C92.Z2
BMH	**ICD-9 procedure**: 41.91<br>**ICD-10 procedure**: 6A550ZV, 6A551ZV<br>**HCPCS**: 38205, 38206, 38230, 38232
BMT	**ICD-9 procedure**: 41.0, 41.00, 41, 41.01, 41.02, 41.03, 41.04, 41.05, 41.06, 41.07, 41.08, 41.09<br>**ICD-10 procedure**: 30230AZ, 30230G0, 30230G1, 30230G2, 30230G3, 30230G4, 30230X0, 30230X1, 30230X2, 30230X3, 30230X4, 30230Y0, 30230Y1, 30230Y2, 30230Y3, 30230Y4, 30233AZ, 30233G0, 30233G1, 30233G2, 30233G3, 30233G4, 30233X0, 30233X1, 30233X2, 30233X3, 30233X4, 30233Y0, 30233Y1, 30233Y2, 30233Y3, 30233Y4, 30240AZ, 30240G0, 30240G1, 30240G2, 30240G3, 30240G4, 30240X0, 30240X1, 30240X2, 30240X3, 30240X4, 30240Y0, 30240Y1, 30240Y2, 30240Y3, 30240Y4, 30243AZ, 30243G0, 30243G1, 30243G2, 30243G3, 30243G4, 30243X0, 30243X1, 30243X2, 30243X3, 30243X4, 30243Y0, 30243Y1, 30243Y2, 30243Y3, 30243Y4, 30250G0, 30250G1, 30250X0, 30250X1, 30250Y0, 30250Y1, 30253G0, 30253G1, 30253X0, 30253X1, 30253Y0, 30253Y1, 30260G0, 30260G1, 30260X0, 30260X1, 30260Y0, 30260Y1, 30263G0, 30263G1, 30263X0, 30263X1, 30263Y0, 30263Y1<br>**HCPCS**: 38240, 38241, 38242, 38243

AML= acute myeloid malignancy; BMH= bone marrow harvest; BMT= bone marrow transplant

© 2021 Wang CY et al. *JAMA Network Open.*

eTable 5. NDC, HCPCS, and CPT Codes for G-CSF Product

GCSF product	HCPCS	NDC
filgrastim	J1440, J1441, J1442	54569482400, 54868252200, 54868252201, 54868305000, 54868502000, 55513020901, 55513020910, 55513020991, 55513034701, 55513034710, 55513034801, 55513034810, 55513053001, 55513053010, 55513054601, 55513054610, 55513092401, 55513092410, 55513092491
tbo-filgrastim	J1446, J1447	63459091001, 63459091011, 63459091012, 63459091015, 63459091017, 63459091036, 63459091201, 63459091211, 63459091212, 63459091215, 63459091217, 63459091236
filgrastim-sndz	Q5101	61314030401, 61314030410, 61314031201, 61314031210
filgrastim-aafi	Q5110	00069029101, 00069029110, 00069029201, 00069029210
Pegfilgrastim and Pegfilgrastim unknown route	J2505	54868522900, 55513019001
pegfilgrastim-OBI	J2505 (CPT 96377 on the same day) from 01/01/2017	55513019201
pegfilgrastim-jmdb	Q5108	67457083306
pegfilgrastim-cbqv	Q5111	70114010101

GCSF= granulocyte-colony stimulating factor; OBI= on-body-injector

© 2021 Wang CY et al. *JAMA Network Open.*

eTable 6. *ICD-9* and *ICD-10* Diagnosis Codes for FN Risk Factors

FN risk factors	ICD-9 diagnosis code	ICD-10 diagnosis code
History of infection	002, 0020, 0021, 0022, 0023, 0029, 003, 0030, 0031, 0032, 00320, 00321, 00322, 00323, 00324, 00329, 0038, 0039, 004, 0040, 0041, 0042, 0043, 0048, 0049, 0080, 00800, 00801, 00802, 00803, 00804, 00809, 0081, 0082, 0083, 0084, 00841, 00842, 00843, 00844, 00845, 00846, 00847, 00849, 0085, 034, 0340, 0341, 035, 036, 0360, 0361, 0362, 0363, 0364, 03640, 03641, 03642, 03643, 0368, 03681, 03682, 03689, 0369, 038, 0380, 0381, 03810, 03811, 03812, 03819, 0382, 0383, 0384, 03840, 03841, 03842, 03843, 03844, 03849, 0388, 0389, 039, 0390, 0391, 0392, 0393, 0394, 0398, 0399, 040, 0400, 0401, 0402, 0403, 0404, 04041, 04042, 0408, 04081, 04082, 04089, 041, 0410, 04100, 04101, 04102, 04103, 04104, 04105, 04109, 0411, 04110, 04111, 04112, 04119, 0412, 0413, 0414, 04141, 04142, 04143, 04149, 0415, 0416, 0417, 0418, 04181, 04182, 04183, 04184, 04185, 04186, 04189, 0419, 101, 112, 1120, 1121, 1122, 1123, 1124, 1125, 1128, 11281, 11282, 11283, 11284, 11285, 11289, 1129, 114, 1140, 1141, 1142, 1143, 1144, 1145, 1149, 115, 1150, 11500, 11501, 11502, 11503, 11504,	A01, A02, A03, A040, A041, A042, A043, A044, A045, A046, A047, A048, A049, A389, J020, J0300, A46, A39, A40, A41, A42, A43, B471, B479, L081, A480, A482, A483, A484, A485, A4851, A4852, A488, K9081, M60009, A493, B95, B96, A690, A601, B370, B378, B371, B377, B375, B376, B38, B39, G02, H32, I32, I39, J17, B40, B41, B480, B42, B43, B449, B450, B452, B453, B457, B458, B459, B46, B470, B481, B482, B483, B484, B488, B49, G00, G01, G042, B451, G02, G06, H440, H050, H6020, H700, I308, I33, I39, J01, J029, J0390, J36, J13, J181, A481, J14, J150, J151, J152, J153, J154, J155, J156, J158, J159, J180, J189, J441, J47, J86, J85, K047, K046, M272, K113, K122, K35, K37, K36, K5712, K5713, K5732, K5733, K61, K65, K67, K6812, K6819, K689, K630, K9402, K9412, K750, K810, N10, N11, N12, N15, N16, N2884, N2885, N2886, N390, N41, N51, O911, L02, L030, K122, L031, L032, L033, L038, L039, L049, L0501, L0502, E832, L080, L088, L0881, L0882, L0889, L089, L88, L980, M00, M726, M462, M463, M86, M896, M908, I96, R6521, R7881, T798, A419, R6520, T826, T827, T836, T845, T846, T847, T857, K6811, T814, T802

© 2021 Wang CY et al. *JAMA Network Open.*

FN risk factors	ICD-9 diagnosis code	ICD-10 diagnosis code
History of infection	11505, 11509, 1151, 11510, 11511, 11512, 11513, 11514, 11515, 11519, 1159, 11590, 11591, 11592, 11593, 11594, 11595, 11599, 116, 1160, 1161, 1162. 117, 1170, 1171, 1172, 1173, 1174, 1175, 1176, 1177, 1178, 1179, 118, 320, 3200, 3201, 3202, 3203, 3207, 3208, 32081, 32082, 32089, 3209, 321, 3210, 3211, 3212, 3213, 3214, 3218, 324, 3240, 3241, 3249, 360, 3600, 36000, 36001, 36002, 36003, 36004, 3601, 36011, 36012, 36013, 36014, 36019, 3602, 36020, 36021, 36023, 36024, 36029, 3603, 36030, 36031, 36032, 36033, 36034, 3604, 36040, 36041, 36042, 36043, 36044, 3605, 36050, 36051, 36052, 36053, 36054, 36055, 36059, 3606, 36060, 36061, 36062, 36063, 36064, 36065, 36069, 3608, 36081, 36089, 3609, 376, 3760, 37600, 37601, 37602, 37603, 37604, 3761, 37610, 37611, 37612, 37613, 3762, 37621, 37622, 3763, 37630, 37631, 37632, 37633, 37634, 37635, 37636, 3764, 37640, 37641, 37642, 37643, 37644, 37645, 37646, 37647, 3765, 37650, 37651, 37652, 3766, 3768, 37681, 37682, 37689, 3769, 38014, 383, 3830, 38300, 38301, 38303, 3831, 3832, 38320, 38321,	T880

© 2021 Wang CY et al. *JAMA Network Open.*

FN risk factors	ICD-9 diagnosis code	ICD-10 diagnosis code
History of infection	38322, 3833, 38330, 38331, 38332, 38333, 3838, 38381, 38389, 3839, 42099, 421, 4210, 4211, 4219, 461, 4610, 4611, 4612, 4613, 4618, 4619, 462, 463, 475, 481, 482, 485, 486, 49121, 494, 4940, 4941, 510, 513, 5225, 5227, 5264, 5273, 5283, 540, 541, 542, 56201, 56203, 56211, 56213, 566, 567, 5695, 56961, 572, 575, 590, 599, 601, 6751, 680, 681, 682, 683, 685, 686, 711, 72886, 730, 7854, 78552, 7907, 9583, 99591, 99592, 9966, 9985, 9993	
History of neutropenia	288.0, 288.00, 288.01, 288.02, 288.03, 288.04, 288.09	D70, D70.0, D70.1, D70.2, D70.3, D70.4, D70.8, D70.9
Renal disease	403.01, 403.11, 403.91, 404.02, 404.03, 404.12, 404.13, 404.92, 404.93, 582.0, 582.1, 582.2, 582.4, 582.8, 582.81, 582.89, 582.9, 583.0, 583.1, 583.2, 583.4, 583.6, 583.7, 583.8, 583.81, 583.89, 583.9, 585.1, 585.2, 585.3, 585.4, 585.5, 585.6, 585.9, 586, 588.0, 588.1, 588.8, 588.81, 588.89, 588.9, V42.0, V45.1, V45.11, V45.12, V56, V56.0, V56.1, V56.2, V56.3, V56.31, V56.32, V56.8	I12.0, I12.0, I13.11, I13.2, N03.2, N03.3, N03.5, N03.8, N08, N03.9, N05.9, N05.2, N05.5, N03.1, N03.4, N03.6, N03.7, N03.0, N03.A, N06.2, N07.2, N05.3, N05.4, N06.3, N06.4, N06.5, N07.3, N07.4, N07.5, E09.21, E09.22, E09.29, M32.14, M32.15, M35.04, N16, N05.0, N05.1, N05.6, N05.7, N06.0, N06.1, N06.6, N06.7, N06.8, N06.A, N07.0, N07.1, N07.6, N07.7, N07.8, N07.A, N14.0, N14.1, N14.2, N14.3, N14.4, N15.0, N15.8, N06.9, N07.9, N15.9, N18.30, N18.31, N18.32
Liver disease	456.0, 456.2, 572.0, 572.1, 572.2, 572.3, 572.4, 572.5, 572.6, 572.7, 572.8, 570, 571.0, 571.1, 571.2, 571.3,	B18.0, B18.1, B18.2, B18.8, B18.9, K73.0, K73.1, K73.2, K73.8, K73.9, K74.0, K74.1, K74.2, K74.3, K74.4, K74.5, K74.6, K74.60, K74.69,

© 2021 Wang CY et al. *JAMA Network Open.*

FN risk factors	ICD-9 diagnosis code	ICD-10 diagnosis code
Liver disease	571.4, 571.40, 571.41, 571.42, 571.49, 571.5, V42.7, 571.6, 571.8, 571.9	K70, K70.0, K70.1, K70.10, K70.11, K70.2, K70.3, K70.9, K70.4, K70.40, K76.6, K76.7, K70.41, K71.3, K71.4, K71.5, K71.7,<br>K72.1, K72.9, K73, K74, K75, K75.0, K75.1, K75.2, K75.3, K75.4, K75.8, K75.81, K75.89, K75.9, K76, K76.0, K76.1, K76.2, K76.3, K76.4, K76.5, K76.8, K76.81, K76.89, K76.9, K70.30, K70.31, I85.0, I85.9, I86.4, I98.2, K70.9, K73.0, K73.1, K73.2, K73.8, K73.9, K74.0, K74.1, K74.2, K70.3, K74.3, K74.4, K74.5, K74.6, K74.60, K74.69
CVD	MI: 410, 410.02, 410.12, 410.22, 410.32, 410.42, 410.52, 410.62, 410.72, 410.82, 410.92, 410.0, 410.00, 410.01, 410.1, 410.10, 410.11, 410.2, 410.20, 410.21, 410.3, 410.30, 410.31, 410.4, 410.40, 410.41, 410.5, 410.50, 410.51, 410.6, 410.60, 410.61, 410.7, 410.70, 410.71, 410.8, 410.80, 410.81, 410.9, 410.90, 410.91, 412<br>PVD: 443.8, 443.81, 443.82, 443.89, 443.9, 444, 444.0, 444.01, 444.09, 444.1,<br>444.2, 444.21, 444.22, 444.8, 444.81, 444.89, 444.9, 451, 451.0, 451.1, 451.11, 451.19, 451.2, 451.8, 451.81, 451.82, 451.83, 451.84, 451.89, 451.9, 452, 453, 453.0-453.4, 453.40-453.42, 453.5, 453.50-453.52,	MI: I21, I21.0, I21.01, I21.02, I21.09, I21.1, I21.11, I21.19, I21.2, I21.21, I21.29, I21.3, I21.4, I25.2<br>PVD: I73, I73.0, I73.00, I73.01, I73.1, I73.8, I73.81, I73.89, I73.9, I74, I74.0, I74.01, I74.09, I74.10, I74.11, I74.19, I74.1-I74.9, I80, I80.0, I80.00, I80.01-I80.03, I80.1, I80.10, I80.11-I80.13, I80.2, I80.201-I80.209, I80.21, I80.211-I80.219, I80.22, I80.221-I80.229, I80.23, I80.231-I80.239, I80.29, I80.291-I80.299, I80.3, I80.8, I80.9, I81, I82, I82.0, I82.1, I82.2, I82.21, I82.210, I82.211, I82.22, I82.220, I82.221, I82.29,<br>I82.290, I82.291, I82.3, I82.4, I82.40, I82.401-I82.409, I82.41, I82.411-I82.419, I82.42, I82.421-I82.429, I82.43, I82.431-I82.439, I82.44, I82.441-I82.449, I82.49,

© 2021 Wang CY et al. *JAMA Network Open.*

FN risk factors	ICD-9 diagnosis code	ICD-10 diagnosis code
CVD	453.6, 453.7, 453.71-453.79, 453.8, 453.81-453.89, 453.9, 557, 557.0, 557.1, 557.9, 415, 415.0, 415.1, 415.11-415.19	I82.491-I82.499, I82.4Y, I82.4Y1-I82.4Y9, I82.4Z, I82.4Z1-I82.4Z9, I82.5, I82.50, I82.501-I82.509, I82.51, I82.511-I82.519, I82.52, I82.521-I82.529, I82.53, I82.531-I82.539, I82.54, I82.541-I82.549, I82.59, I82.591-I82.599, I82.5Y, I82.5Y1-I82.5Y9, I82.5Z, I82.5Z1-I82.5Z9, I82.6, I82.60, I82.601-I82.609, I82.61, I82.611-I82.619, I82.62, I82.621-I82.629, I82.7, I82.70, I82.701-I82.709, I82.71, I82.711-I82.719, I82.72, I82.721-I82.729, I82.8, I82.81, I82.811-I82.819, I82.89, I82,890, I828.91, I82.9, I82.90, I82.91, I82.A, I82.A1, I82.A11-I82.A19, I82.A2, I82.A21-I82.A29, I82.B, I82.B1, I82.B11-I82.B19, I82.B2, I82.B21-I82.B29, I82.C, I82.C1, I82.C11-I82.C19, I82.C2, I82.C21-I82.C29, K55.0, K55.1, K55.9, I26, I26.0 I26.01-I26.09, I26.9, I26.90-I26.99<br>Stroke: I60, I60.0, I60.00-I60.02, I60.1, I60.10-I60.12, I60.2, I60.20-I60.22, I60.3, I60.30-I60.32, I60.4, I60.5, I60.50-I60.52, I60.6-I60.9, I61,<br>I61.0-I61.9, I63, I63.0, I63.00-I63.019, I63.02, I63.03, I63.031-I63.039, I63.09, I63.1, I63.10, I63.11, I63.111-I63.119, I63.12, I63.13, I63.131-I63.139, I63.19, I63.2, I63.20, I63.21, I63.211-I63.219, I63.22, I63.23, I63.231-I63.239, I63.29, I63.3, I63.30, I63.31, I63.311-I63.319, I63.32,

© 2021 Wang CY et al. *JAMA Network Open.*

FN risk factors	ICD-9 diagnosis code	ICD-10 diagnosis code
CVD	Stroke: 430, 431, 433.00-433.91, 434.00, 434.01, 434.10, 434.11, 434.90,<br>434.91, 435, 435.0-435.3, 435.8, 435.9, 436, 437, 437.0-437.9, 438, 438.0, 438.1, 438.10-438.14, 438.19,<br>438.2, 438.20-438.22, 438.3, 438.30-438.32, 438.4, 438.40-438.42, 438.5, 438.50-438.53, 438.6, 438.7, 438.8, 438.81-438.85, 438.89, 438.9<br>HF: 402.01, 402.11, 402.91, 398.91, 404.01, 404.03, 404.11, 404.13, 404.91, 404.93, 428, 428.0, 428.1,<br>428.2, 428.20, 428.21, 428.22, 428.23, 428.3, 428.30, 428.31, 428.32, 428.33, 428.4, 428.40, 428.41, 428.42, 428.43, 428.9	I63.321-I63.329, I63.33, I63.331-I63.339, I63.34,<br>I63.341-I63.349, I63.39, I63.4, I63.40, I63.41, I63.411-I63.419, I63.42, I63.421-I63.429, I63.43, I63.431-I63.439, I63.44, I63.441-I63.449, I63.49, I63.5, I63.50, I63.51, I63.511-I63.519, I63.52, I63.521-I63.529, I63.53, I63.531-I63.539, I63.54, I63.541-I63.549, I63.59, I63.6, I63.8, I63.9, I67.0, I67.81-I67.83, I67.89, 167.9<br>HF: I11.0, I09.81, I50, I50.2, I50.20, I50.21, I50.22, I50.23, I50.3, I50.30, I50.31, I50.32, I50.33, I50.4, I50.40, I50.41, I50.42, I50.43, I50.8, I50.81, I50.810, I50.811, I50.812, I50.813, I50.814, I50.82, I50.83,<br>I50.84, I50.89, I50.9, I97.13, I97.130, I97.131, T86.22, T86.32, I13.0, I13.2
Diabetes	250.00, 250.02, 250.10, 250.12, 250.20, 250.22, 250.30, 250.32, 250.4,<br>250.40, 250.41, 250.42, 250.43, 250.50, 250.52, 250.60, 250.62, 250.70, 250.72, 250.80, 250.82, 250.90, 250.92	E11, E11.0, E11.00, E11.01, E11.3, E11.31, E11.311, E11.319, E11.32, E11.321, E11.329, E11.33, E11.331, E11.339, E11.34, E11.341, E11.349, E11.35, E11.351, E11.359, E11.36, E11.39, E11.4, E11.40, E11.41, E11.42, E11.43, E11.44, E11.49, E11.5, E11.51, E11.52, E11.59, E11.6, E11.61, E11.610, E11.618, E11.62, E11.620, E11.621, E11.622, E11.628, E11.63, E11.630, E11.638, E11.64, E11.641, E11.649, E11.65, E11.69, E11.8, E11.9, E10.22, E11.22, E11.2,

© 2021 Wang CY et al. *JAMA Network Open.*

FN risk factors	ICD-9 diagnosis code	ICD-10 diagnosis code
Diabetes		E11.21, E11.29
COPD	416.8, 416.9, 506.4, 508.1, 508.8	I27.8, I27.81, I27.82, I27.89, I27.9, J40, J41, J41.0, J41.1, J41.8, J42, J43, J43.0, J43.1, J43.2, J43.8, J43.9, J44, J44.0, J44.1, J44.9, J47.0, J47.1, J47.9, J60, J61, J62, J62.0, J62.8, J63, J63.0, J63.1, J63.2, J63.3, J63.4, J63.5, J63.6, J64, J66, J66.0, J66.1, J66.2, J66.8, J67, J67.0, J67.1, J67.2, J67.3, J67.4, J67.5, J67.6, J67.7, J67.8, J67.9, J68.4, J70.1, J70.3
HIV/AIDS	042, 042.0, 042.1, 042.2, 042.9, 043, 043.1, 043.2, 043.3, 043.9, 044, 044.0,<br>044.9	B20, B20.0, B20.1, B20.2, B20.3, B20.4, B20.5, B20.6, B20.7, B20.8, B20.9, B21, B21.0, B21.1, B21.2, B21.3, B21.7, B21.8, B21.9, B22, B22.0, B22.1, B22.2, B22.7, B23.0, B23.1, B23.2, B23.8, B24, B97.35, O98.7, O98.71, O98.711, O98.712, O98.713, O98.719, O98.72, O98.73, Z21
Metastasis	196, 1960, 1961, 1962, 1963, 1965, 1966, 1968, 1969, 197, 1970, 1971, 1972, 1973, 1974, 1975, 1976, 1977, 1978, 198, 1980, 1981, 1982, 1983, 1984, 1985, 1986, 1987, 1988, 19881, 19882, 19889, 2097, 20970, 20971, 20972, 20973, 20974, 20975, 20979, 78951	C770, C771, C772, C773, C774, C775, C778, C779, C7800, C7801, C7802, C781, C782, C7830, C7839, C784, C785, C786, C787, C7880, C7889, C7900, C7901, C7902, C7910, C7911, C7919, C792, C7931, C7932, C7940, C7949, C7951, C7952, C7960, C7961, C7962, C7970, C7971, C7972, C7981, C7982, C7989, C799, C7B00, C7B01, C7B02, C7B03, C7B04, C7B09, C7B1, C7B8, C800, C801, R180
Metastatic cancer to bone	198.5, 209.73	C7951, C7952, C7B03

© 2021 Wang CY et al. *JAMA Network Open.*

eTable 7. Cancer Incidence by Site in Overall US Population, Commercial Insurance, and Medicare FFS Database

	Overall US population†	IBM MarketScan‡	Medicare FFS database‡
Breast cancer, rate per 100,000			
2014	129.2	129.4	363.5
2015	130.6	124.6	350.1
2016	128.9	119.3	347.0
2017	131.1	111.5	361.9
2018	131.3	106.0	362.2
2019	--	105.3	--
Lung cancer, rate per 100,000			
2014	55.6	54.1	270.2
2015	55.0	49.4	267.9
2016	53.8	46.1	263.5
2017	53.1	42.0	268.9
2018	50.8	36.0	271.1
2019	--	32.7	--
Non-Hodgkin lymphoma, rate per 100,000			
2014	20.3	39.1	122.3
2015	20.1	37.1	134.0
2016	20.0	34.2	120.8
2017	19.6	31.5	127.5
2018	19.4	29.6	137.3
2019	--	28.1	--
Colorectal cancer, rate per 100,000			
2014	39.5	56.7	222.8
2015	38.7	52.7	215.6
2016	38.3	50.6	194.9
2017	37.4	45.4	205.1
2018	36.8	41.0	211.6
2019	--	40.1	--

† Rates are per 100,000 and are age-adjusted to the 2000 US standard population obtained from SEER*Explorer. ‡ cancer incidence in both databases are estimated using diagnosis codes presented in eTable 3 based on 1 inpatient or 2 outpatient claims (within 90 days) during the year. Incidence are calculated as follow: *Beneficiaries who have complete continuous enrollment during the year and the year before as well as cancer diagnosis during the year without cancer diagnosis in the year before/ beneficiaries who have complete continuous enrollment during the year and the year before without cancer diagnosis in the year before*

© 2021 Wang CY et al. *JAMA Network Open.*

eTable 8. Year of Chemotherapy Initiation and Patient Characteristics Associated with Use of G-CSF by Regimen FN Risk Category Among Patients Younger Than 65 Years in the Commercially Insured Population

**Patient characteristics**	**Adjusted odds ratio (95% confidence interval)**			
**Regimen FN risk level**	**All**	**High**	**Intermediate**	**Low**
Sample size	66,891	32,330	21,780	12,781
Year of Chemotherapy Initiation				
2014	ref	ref	ref	ref
2015	0.95 (0.89-1.01)	1.04 (0.96-1.13)	0.82* (0.74-0.92)	0.94 (0.81-1.09)
2016	0.85* (0.80-0.91)	0.92* (0.85-1.00)	0.78* (0.70-0.87)	0.77* (0.66-0.90)
2017	0.91* (0.85-0.97)	1.09* (1.00-1.19)	0.70* (0.63-0.79)	0.78* (0.66-0.92)
2018	0.93* (0.87-0.99)	1.31* (1.19-1.43)	0.63* (0.56-0.71)	0.64* (0.54-0.76)
2019	1.02 (0.95-1.09)	1.58* (1.43-1.75)	0.59* (0.53-0.67)	0.76* (0.64-0.91)
Age				
<45	ref	ref	ref	ref
45-54	1.08* (1.03-1.15)	1.12* (1.04-1.21)	1.16* (1.02-1.32)	0.90 (0.74-1.09)
55-64	1.13* (1.07-1.19)	1.04 (0.97-1.12)	1.39* (1.23-1.56)	1.02 (0.85-1.22)
Female vs male	1.39* (1.33-1.46)	1.83* (1.67-2.01)	1.19* (1.11-1.28)	1.24* (1.12-1.37)
Regimen FN risk level				
High	16.14* (15.12-17.22)	N/A	N/A	N/A
Intermediate	1.49* (1.40-1.58)	N/A	N/A	N/A
Low	ref	N/A	N/A	N/A
Risk factors for FN				
History of chemotherapy	0.63* (0.57-0.70)	0.45* (0.34-0.60)	0.91 (0.80-1.05)	0.47* (0.40-0.55)
History of radiation therapy	0.73* (0.68-0.78)	0.82* (0.70-0.97)	0.66* (0.60-0.72)	0.83* (0.73-0.96)

© 2021 Wang CY et al. *JAMA Network Open.*

Patient characteristics	Adjusted odds ratio (95% confidence interval)			
**Regimen FN risk level**	**All**	**High**	**Intermediate**	**Low**
Recent surgery	0.86* (0.82-0.89)	0.93* (0.88-0.98)	0.63* (0.59-0.68)	1.23* (1.10-1.37)
History of infection	0.99 (0.95-1.03)	0.97 (0.91-1.04)	1.00 (0.93-1.07)	1.06 (0.96-1.17)
History of neutropenia	3.92* (3.65-4.22)	3.33* (3.00-3.69)	4.87* (4.32-5.50)	3.83* (3.32-4.42)
Renal disease	0.87 (0.73-1.05)	0.93 (0.70-1.23)	0.81 (0.62-1.06)	0.83 (0.56-1.23)
Liver disease	0.95 (0.90-1.01)	1.04 (0.94-1.16)	0.99 (0.91-1.08)	0.96 (0.84-1.11)
CVD	1.09* (1.02-1.16)	1.01 (0.91-1.13)	1.18* (1.07-1.29)	1.04 (0.92-1.18)
Diabetes	1.05* (1.00-1.11)	1.04 (0.96-1.13)	1.08 (0.99-1.18)	1.04 (0.91-1.18)
COPD	1.30* (1.20-1.41)	1.05 (0.89-1.23)	1.63* (1.46-1.82)	1.14 (0.99-1.33)
HIV/AIDS	0.87 (0.64-1.18)	0.99 (0.61-1.60)	0.59* (0.36-0.96)	1.76 (0.90-3.42)
Metastasis	1.07* (1.03-1.12)	1.23* (1.16-1.30)	0.85* (0.79-0.92)	0.84* (0.75-0.93)
Metastatic cancer to bone	1.24* (1.14-1.34)	0.68* (0.56-0.81)	1.47* (1.33-1.62)	1.17* (1.00-1.36)

© 2021 Wang CY et al. *JAMA Network Open.*

eTable 9. Year of Chemotherapy Initiation and Patient Characteristics Associated with Use of G-CSF by Regimen FN Risk Category Among Patients Aged 65 Years or Older in Medicare Population

**Patient characteristics**	**Adjusted odds ratio (95% confidence interval)**			
**Regimen FN risk level**	**All**	**High**	**Intermediate**	**Low**
Sample size	28,566	6,653	11,638	10,275
Year of Chemotherapy Initiation				
2014	ref	ref	ref	ref
2015	1.12 (0.96-1.31)	1.06 (0.78-1.43)	1.14 (0.92-1.43)	1.13 (0.87-1.47)
2016	1.13 (1.00-1.28)	1.32* (1.02-1.70)	1.06 (0.88-1.27)	1.06 (0.85-1.31)
2017	1.17* (1.04-1.32)	1.62* (1.27-2.07)	1.06 (0.89-1.27)	1.02 (0.83-1.25)
2018	1.15* (1.01-1.30)	1.88* (1.47-2.41)	0.97 (0.81-1.16)	1.04 (0.84-1.28)
Age				
65-69	ref	ref	ref	ref
70-74	1.06 (0.98-1.14)	0.90 (0.76-1.06)	1.12* (1.00-1.25)	1.11 (0.97-1.27)
75-80	1.11* (1.03-1.20)	0.89 (0.73-1.07)	1.19* (1.06-1.33)	1.21* (1.06-1.40)
>80	1.01 (0.92-1.11)	1.00 (0.78-1.29)	1.07 (0.93-1.23)	1.04 (0.88-1.21)
Female vs male	1.04 (0.98-1.11)	1.20 (1.00-1.43)	1.02 (0.93-1.11)	1.03 (0.93-1.14)
Regimen FN risk level				
High	17.73* (16.17-19.44)	N/A	N/A	N/A
Intermediate	1.54* (1.44-1.65)	N/A	N/A	N/A
Low	ref	N/A	N/A	N/A
Risk factors for FN				
History of chemotherapy	0.47* (0.42-0.53)	0.51* (0.36-0.71)	0.87 (0.73-1.03)	0.29* (0.24-0.34)
History of radiation therapy	0.60* (0.56-0.65)	0.47* (0.36-0.61)	0.55* (0.50-0.61)	0.83* (0.71-0.96)

© 2021 Wang CY et al. *JAMA Network Open.*

Patient characteristics	Adjusted odds ratio (95% confidence interval)			
**Regimen FN risk level**	**All**	**High**	**Intermediate**	**Low**
Recent surgery	0.97 (0.91-1.03)	1.14 (0.99-1.31)	0.76* (0.69-0.83)	1.33* (1.19-1.49)
History of infection	1.10* (1.03-1.17)	1.03 (0.88-1.20)	1.16* (1.06-1.27)	1.06 (0.95-1.18)
History of neutropenia	3.93* (3.57-4.32)	2.14* (1.75-2.63)	4.64* (4.02-5.35)	4.15* (3.57-4.82)
Renal disease	0.97 (0.80-1.16)	0.86 (0.58-1.27)	1.16 (0.91-1.48)	0.85 (0.61-1.18)
Liver disease	0.93 (0.80-1.16)	0.98 (0.79-1.21)	0.97 (0.87-1.07)	0.93 (0.81-1.07)
CVD	0.99 (0.93-1.06)	0.89 (0.75-1.05)	1.03 (0.94-1.13)	0.97 (0.87-1.08)
Diabetes	1.00 (0.94-1.07)	0.87 (0.75-1.01)	1.05 (0.96-1.15)	0.98 (0.87-1.08)
COPD	1.26* (1.18-1.35)	0.87 (0.72-1.05)	1.51* (1.37-1.66)	1.16* (1.03-1.30)
HIV/AIDS	0.16* (0.13-0.21)	0.96 (0.55-1.68)	0.08* (0.05-0.11)	0.26* (0.16-0.41)
Metastasis	0.93 (0.87-1.00)	1.03 (0.88-1.21)	0.89* (0.80-0.99)	0.82* (0.73-0.92)
Metastatic cancer to bone	1.50* (1.37-1.63)	0.77 (0.55-1.07)	1.63* (1.46-1.82)	1.29* (1.09-1.52)

© 2021 Wang CY et al. *JAMA Network Open.*
